# Multi-AUV autonomous task planning based on the scroll time domain quantum bee colony optimization algorithm in uncertain environment

**DOI:** 10.1371/journal.pone.0188291

**Published:** 2017-11-29

**Authors:** Jianjun Li, Rubo Zhang, Yu Yang

**Affiliations:** 1 College of Computer Science and Technology, Harbin Engineering University, Harbin, China; 2 School of Computer and Information Engineering, Harbin University of Commerce, Harbin, China; 3 College of Electromechanical & Information Engineering, Dalian Nationalities University, Liaoning Dalian, China; Southwest University, CHINA

## Abstract

Research on distributed task planning model for multi-autonomous underwater vehicle (MAUV). A scroll time domain quantum artificial bee colony (STDQABC) optimization algorithm is proposed to solve the multi-AUV optimal task planning scheme. In the uncertain marine environment, the rolling time domain control technique is used to realize a numerical optimization in a narrowed time range. Rolling time domain control is one of the better task planning techniques, which can greatly reduce the computational workload and realize the tradeoff between AUV dynamics, environment and cost. Finally, a simulation experiment was performed to evaluate the distributed task planning performance of the scroll time domain quantum bee colony optimization algorithm. The simulation results demonstrate that the STDQABC algorithm converges faster than the QABC and ABC algorithms in terms of both iterations and running time. The STDQABC algorithm can effectively improve MAUV distributed tasking planning performance, complete the task goal and get the approximate optimal solution.

## Introduction

The Autonomous Underwater Vehicle Task Planning (AUVTP) is based on the AUV task, AUV task load and quantity, pre-setting and coordinating the specific combat mission. Task planning includes task allocation, route planning, sensor planning and link planning. From time division, AUVTP can be divided into real-time planning and pre-planning. Real-time planning is a viable route for AUV during the voyage, based on actual navigation conditions and subsea environmental changes, including options for pre-planned revisions and contingency plans[1,2]. Pre-planning is AUV pre-sailing to develop, comprehensive mission requirements, the marine environment and communication conditions and other information, the development of long-term mission planning. Real-time mission planning, due to the process of navigation, emphasizes real-time planning capabilities in dynamic, uncertain battlefield environments, and faces more complex and greater challenges than pre-planning.

In the high combat environment, the battlefield information changes, the need for AUV to reduce the manual participation and reduce the dependence on other systems, can independently adjust the program and re-planning, to achieve truly intelligent self-planning. Intelligent autonomous planning is the further development of artificial intelligence technology, is the ultimate goal of mission planning. At present, some local independent planning and control technology has made some development, such as autonomous search, autonomous target recognition, autonomous fire distribution and so on [3–5].

Beyond achieving various underwater applications, the motion control of AUV is one of the essential problems in attaining underwater operational objectives [6,7]. However, the modelling parameters of the underwater vehicle is difficult to be accurately acquired and the vehicle is also vulnerable by environmental disturbances including ocean currents and waves[8]. These internal and external uncertainties along with the nonlinearity of the vehicle dynamics render the AUV control problem difficult.

With the rapid development of swarm intelligence algorithms, many researchers have simulated insect foraging behavior and have introduced a response threshold model to assign tasks; other experts have introduced the ant colony algorithm to solve the large-scale task allocation problem based on the time series [9–11]. Some experts have also designed a task model and proposed an improved discrete particle swarm optimization algorithm to solve the problem [12,13]. These methods provide a new way to solve the problem of allocating tasks among multiple robots. The present paper explores multi-AUV autonomous task planning, especially the rolling time domain AUV mission planning in uncertain environment, and use the bionic task planning method of quantum bee optimization algorithm to explore the AUV autonomous mission planning theory.

## Quantum bee colony optimization algorithm

### Quantum artificial bee colony algorithm

The bee colony optimization algorithm is a type of meta-heuristic optimization method to imitate the behavior of natural bees. Ferrante et al. [14] proposed a self-organization model, which was applied to task partitioning. Grozinger [15] proposed a self-organizing model, which showed the communication in the bee colony through many methods, including "swing dance" and odor. This self-organization model can complete different tasks in different social classes. Karaboga et al. [16] successfully applied the colony algorithm to the problem of function extremum optimization and systematically introduced the artificial bee colony (ABC) model. Civicioglu and Besdok [17] analyzed a conceptual comparison of the Cuckoo search, particle swarm optimization, differential evolution and artificial bee colony algorithms. Loubière et al [18] proposed a sensitivity analysis method for driving the artificial bee colony algorithm’s search process, a new approach to random selection in neighborhood search. Quantum calculation is a new computational method based on quantum mechanics theory. Quantum Artificial Bee Colony Algorithm (QABC) uses the quantum revolving door to realize the optimal position search of the bee, and uses the qubit to encode the current position of the bee. The quantum bezel is used to realize the variation of the bee position and avoid premature convergence Phenomenon occurs.

In the quantum space, the particle state $ih\frac{\lambda }{\lambda t}\varphi (X,t)=H\varphi (X,t)$ is represented by the wave function *φ*(*X*,*t*), where H is the Hamiltonian operator and *h* is Planck’s constant. If the particle undergoes a one-dimensional potential well movement at the center point of *Q*, the position determined by the stochastic equation is $X=Q\pm \frac{h^{2}}{2m\gamma }\ln(1/u)$, where m is the particle mass and *u* is a random number in the interval of (0, 1) [19].

Thus, we can obtain a formula of the quantum bee colony optimization algorithm:

$$X_{i,j}(t+1)=Q_{i,j}(t)\pm \lambda \left|X_{i,j}(t)-X_{i\neq j,j}(t)\right|\ln(1/u_{i,j}(t))$$ (1)

In the formula, i is the bee number, j is the dimension, *X*_*i*,*j*_ is the bee optimization position, and *λ* is a constant. In addition,

$$Q_{i,j}(t)=\alpha_{j}(t)\times Q_{i,j}(t)+(1-\varphi_{j})\times G_{j}(t)$$ (2)

In the formula, *α*_*j*_ is a random number in the interval of (0, 1), *Q*_*i*,*j*_(*t*) is the best estimate of the current position of an individual bee, and *G*_*j*_(*t*) is the best estimate of the current position of all bees.

The best estimate of the position of the i-th bee is

$$Q_{i}(t)=\left\{ \begin{array}{ll} X_{i}(t) & \;f\left[X_{i}(t)\right]<f\left[Q_{i}(t-1)\right]\\ Q_{i}(t-1) & \;f\left[X_{i}(t)\right]\geq f\left[Q_{i}(t-1)\right] \end{array}\right.$$ (3)

The best estimate of the global position is determined by $g=\arg\;\underset{1\leq i\leq m}{\min}\left\{f\left[Q_{i}(t)\right]\right\}$ and *G*(*t*) = *Q*_*g*_(*t*).

### Task allocation model based on quantum bee colony optimization algorithm

The managers are denoted by AUV_*α*_ in the distributed contract net. They are responsible for managing the task, and the other AUV_*i*_ are responsible for bidding the task. The task allocation process includes four steps: task bidding, bid, bid winning and task execution based on the contract net. The contract net task allocation model based on the differential evolution quantum bee colony algorithm is as follows:

Assume that there are *N*_*V*_ AUVs, $Task=\left\{Task_{1},Task_{2},\cdots ,Task_{N_{M}}\right\}$, the number of task targets is *N*_*M*_, $V=\left\{V_{1},V_{2},\cdots ,V_{N_{V}}\right\}$, the number of AUVs is *N*_*V*_, $Menace=\left\{Menace_{1},Menace_{2},\cdots ,Menace_{N_{Q}}\right\}$, and the number of threat sources is *N*_*Q*_. The AUVs, task targets, and threat sources can include many types. If the same type of task is performed by different AUVs, the implementation effect is different. Assuming that the task set assigned to *AUV*_*i*_ is $T_{i}=\left\{Task_{i}^{1},Task_{i}^{2},\cdots ,Task_{i}^{n_{i}}\right\}$, the multi-AUV distributed task allocation problem can be translated as follows: Assign the existing tasks to multiple AUVs in the shortest possible time, i.e., $\overset{N_{V}}{\underset{i=1}{\cup }}T_{i}=Task$; each AUV has only one task, i.e., ∀*i*,*j* ∈ {1,⋯,*N*_*V*_}, *i* ≠ *j*, and *T*_*i*_∩*T*_*j*_ = ∅. If the maximum number of tasks executed by the multi-AUV system is less than the number of tasks that should be allocated, the assignment can be optimized to improve the overall efficiency of the multi-AUV task allocation system according to the following objectives.

Objective one: To maximize the overall effectiveness $\sum_{i=1}^{N_{V}}\theta_{i}(T_{i})$ of the AUV after finishing the task, *θ*_*i*_(*T*_*i*_) is the performance after the task set *T*_*i*_ is completed by *V*_*i*_.

Objective two: To minimize the required time max_*i*∈*V*_ *Time*_*i*_(*T*_*i*_) of the task to be completed by the AUV, *Time*_*i*_(*T*_*i*_) is the time at which the task set *T*_*i*_ is finished by *V*_*i*_.

Objective three: To balance the task load of each AUV, $\sum_{i=1}^{N_{V}}\left|Tload_{i}(T_{i})-\bar{Tload}\right|$ is minimized, where *Tload*_*i*_(*T*_*i*_) is the task load of *V*_*i*_ and $\bar{Tload}$ is the average task load for each AUV.

## Multi-AUV autonomous task planning in uncertain environment

### Scroll time domain task allocation model

Scroll time domain control is a numerical optimization problem in a reduced time range. For multi-AUV applications, rolling time domain control is one of the better task planning techniques. In an uncertain environment, since the sensor information can be embedded in the online solution, the task execution can be handled very well; at the same time, because only the local information is embedded, the calculated workload can be greatly reduced. The cost function is weighed between the AUV dynamics state, the environment and the cost, and the final cost function is submitted to the online task planning to ensure that the task objectives are completed and the approximate optimal solution is obtained.

The scroll time domain task assignment is a given task set *T*, The distance between tasks *d*(*i*,*j*), And lists the specified length *L*_*c*_ the sequence of tasks in memory[20, 21]. The estimate of each task sequence is

$$R_{VP}=\sum \lambda T_{ip}R_{wd}$$ (4)

Where *T*_*ip*_ is the time at which task *p* is completed in task sequence *i*, *R*_*wd*_ is the assigned task weight, and *λ* is the time scale factor.

When the values of all task sequences *R*_*VP*_ are given, the scrolling time domain task allocation algorithm can select the optimal task sequence for AUV.

$$\begin{array}{l} \max\;L=\sum_{P=1}^{N_{VP}}\sum_{V=1}^{N_{V}}R_{VP}X_{VP}\\ \sum_{V=1}^{N_{V}}\sum_{P=1}^{N_{VP}}A_{VP}X_{VP}\leq 1(X_{VP}\in \left\{0,1\right\})\\ \sum_{p=1}^{N_{VP}}x_{vp}\leq 1(\forall v\in 1,\cdots ,N_{v}) \end{array}$$ (5)

Where *x*_*vp*_ is a binary variable, its value is 1 when the *P*^*th*^ task sequence is selected, otherwise its value is 0; when task *P* in the task sequence *i* is searched, the value of *A*_*VP*_ is 1, otherwise it is 0.

Time adjustment parameter setting, parameter *λ*_*et*_ indicates *K*^*th*^ update task arrival time estimation value, completion time of task time depends on test state time *T*_*c*_, *h* is the time value given by the bottom clock. If *λ*_*et*_ > *δ*_min_, it will be updated, where *δ*_min_ is a relatively fixed user fixed value.

$$\lambda_{et}=\left|T^{k}-T_{c}-h\right|$$ (6)

Space adjustment parameter setting, *X*_*t*_ for the reference configuration; *X* for the measured configuration value.

$$\lambda_{s}=\left|X_{t}-X\right|$$ (7)

If *λ*_*s*_ > *α*_min_, it will be updated, and parameters *δ*_min_ and *α*_min_ affect the number of updates.

### Multi-AUV distributed task allocation with balanced coefficient

To enable multiple AUVs to quickly complete the task and achieve global optimization, first, the task is distributed to the entire AUV team with the smallest cost using the contract net to ensure the global optimization of task implementation. Then, the balance coefficient is used to make the entire AUV team distribute and achieve the tasks in the shortest time.

The balanced coefficient $B_{eq}^{R}$ is introduced in the contract net distributed robot task allocation. Each robot uses its cost function to count the workload: the workload is the cost of robot R in the entire process of the work. Each robot broadcasts its workload to the entire team and calculate its *B*_*eq*_. The formula of the balance coefficient for robot R is as follows:

$$B_{eq}^{R}=\frac{wa(R)-\bar{wa}}{\bar{wa}}$$ (8)

where $\bar{wa}$ is the average workload of all robots in the team.

$B_{eq}^{R}$<0: robot R has a lighter workload than the other robots;
$B_{eq}^{R}$>0: robot R has a heavier workload than the other robots;
$B_{eq}^{R}$>$B_{eq}^{R1}$ 0: robot R has a heavier workload than robot R1;

In the contract net, the robot can take the task at the minimum cost, and the workload to be obtained should not be excessive. Thus, the task can be estimated from the balance coefficient *B*_*eq*_. The formula of the task is estimated by robot R as follows:

$$rt^{'R}(T_{1})=rt^{R}(T_{1})-B_{eq}^{R}\times \left|rt^{R}(T_{1})\right|$$ (9)

The task can be estimated using the balance coefficient *B*_*eq*_ of robot R. The following effects can be obtained:

1. A robot with a larger workload cannot easily obtain new tasks, and its tasks are more likely to be reassigned because its task utility is low.
2. A robot with a smaller workload easily obtains new tasks and does not easily give up its task because its task utility is high.

### AUV autonomous learning mechanism

AUV has an autonomous learning mechanism that needs to include an implementation component, Multi-Level Executive (MLE) and Deliberative Layer (DL). Including two major execution instruction mechanisms.

First, the dynamic task is inserted. Insert the new task into the relative position that has been ranked in the task sequence according to the insertion model.

Second, the dynamic task is terminated. If the task has not yet started running, the termination of the task is canceled. If the task has already started running, the termination of the task is interrupted.

Multi-AUV system flexibility and openness, multi-AUV system can be combined with the group of swarm intelligence algorithm. AUV as an agent can not only interact with the optimal AUV in the current population, but also can self-study in multiple iterations to complete the accumulation of knowledge, so as to improve the ability to solve the problem, to achieve group intelligence optimization.

Let the position of *AUV* S_*ij*_ in the solution space be *S*_*i*,*j*_ = (*s*_1_,*s*_2_,⋯,*s*_*n*_), the size of the solution space is m*S*_*size*_ × m*S*_*size*_, and the position formula of each *AUV* mS_*i*'*j*'_(*i*',*j*' = 1,2,⋯,*mS*_*size*_) is:

$$mS_{i'j'}=\left\{ \begin{array}{ll} S_{i,j} & \;i'=1,j'=1\\ SS_{i',j'} & \;\mathrm{Other} \end{array}\right.$$ (10)

Formula *SS*_i',*j*'_ = (*SS*_*i*',*j*',1_*SS*_*i*',*j*',2_⋯*SS*_*i*',*j*',n_), where *SS*_*i*',*j*',*n*_ is:

$$SS_{i',j',n}=\left\{ \begin{array}{l} L_{n\min},\;S_{n}rand(1-mR,1+mR)<L_{n\min}\\ L_{n\max},\;S_{n}rand(1-mR,1+mR)>L_{n\max}\\ S_{n}rand(1-mR,1+mR),\;\mathrm{Other} \end{array}\right.$$ (11)

In the formula, *mR* is the local search radius,and *mR* ∈ [0,1], *rand*(1−*mR*1+m*R*) is a random number of (1−*mR*1+m*R*).

Because AUV has the ability of autonomous learning mechanism, so as long as the optimal state of each iteration process of self-learning, you can achieve their own task control ability to improve. This not only improves the efficiency of the algorithm, but also re-searches the current optimal state to improve the accuracy of the search algorithm.

## Experimental results and analysis

### Experimental parameter assignment

To evaluate the performance of the Multi-AUV autonomous task planning based on the scroll time domain quantum bee colony optimization algorithm in uncertain environment. The simulation experiment were performed on a laptop computer which has a dual core 3.2 GHz CPU and 8 GB RAM using MATLAB. The MATLAB parallel computing toolbox is used to execute the algorithm for all clusters in parallel. The conditions of the simulation experiment are as follows:

A set of thirty task items to be assigned is selected. The thirty tasks can be divided into three categories: *T*_1_, *T*_2_, and *T*_3_. *AUV*_1_, *AUV*_2_, and *AUV*_3_ are involved in the bidding of the AUVs and all tasks of the bid. The bid value of the completed task, trust and initial ability are shown in Table 1. The influence factors of the AUV load, ability and trust degree are 0.5, 0.3and 0.2, respectively, in the bidding strategies of the contract net task allocation based on the scroll time domain quantum bee colony algorithm.

**Table 1:** Initial value of the completed task, trust and ability.

	*T*_1_			*T*_2_			*T*_3_		
	Bidding value	Trust degree	Ability	Bidding value	Trust degree	Ability	Bidding value	Trust degree	Ability
*AUV*_1_	3	0.7	0.8	2	0.9	0.9	2	0.8	0.7
*AUV*_2_	4	0.8	0.6	3	0.8	0.8	3	0.7	0.9
*AUV*_3_	5	0.6	0.7	3	0.7	0.7	4	0.6	0.8

The simulation experiment has 2 objectives. When the bidding and tendering stage are identical, the first objective is to test and compare the contract net model based on the scroll time domain quantum bee colony algorithm and the traditional contract net model. The second objective is to compare the performance in four aspects: efficiency of task allocation, average AUV load, number of bid AUV allocated tasks, and proportion relation of the corresponding type of task ability.

### Experimental verification

**Fig 1:**
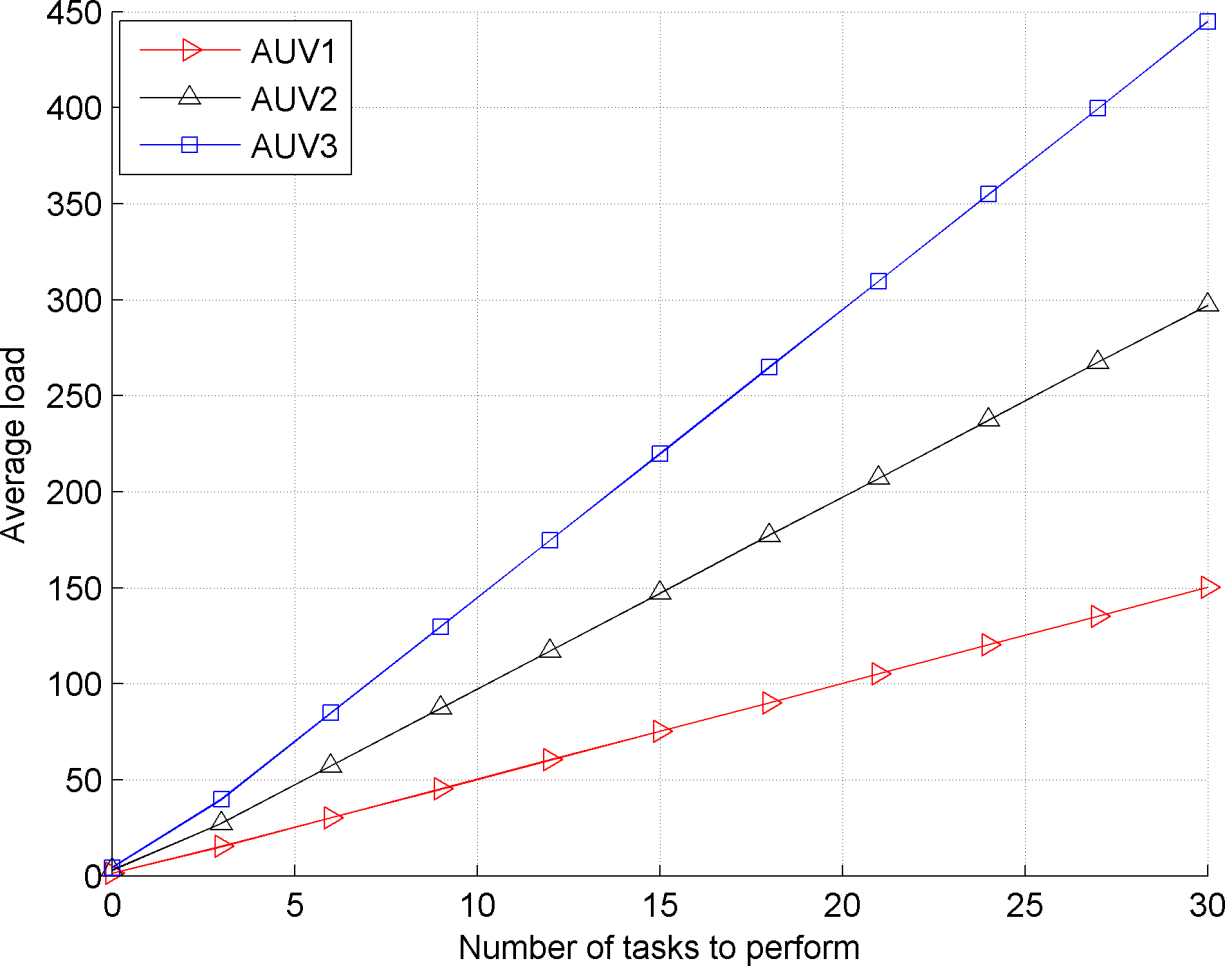
Average load of *AUV*_1_, *AUV*_2_ and *AUV*_3_ in the traditional contract net model *AUV*_2_ *AUV*_3_.

**Fig 2:**
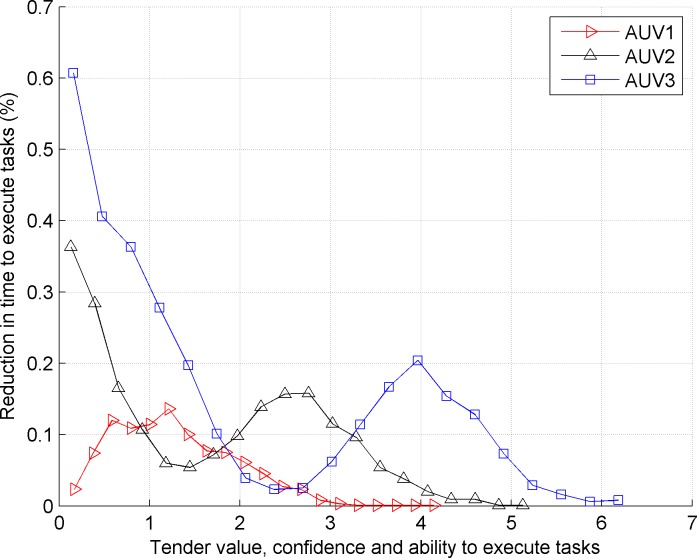
Reduced proportion (%) of *AUV*_1_, *AUV*_2_ and *AUV*_3_ *AUV*_2_ *AUV*_3_ when they executed tasks in the traditional contract net model.

After the experiment, the simulation results are as follows. Fig 1 shows the average load of *AUV*_1_, *AUV*_2_ and *AUV*_3_ in the traditional contract net model *AUV*_1_ *AUV*_2_ *AUV*_3_. Fig 2 shows the reduced proportion (%) when *AUV*_1_, *AUV*_2_ and *AUV*_3_ *AUV*_2_ *AUV*_3_ execute tasks in the traditional contract net model.

**Fig 3:**
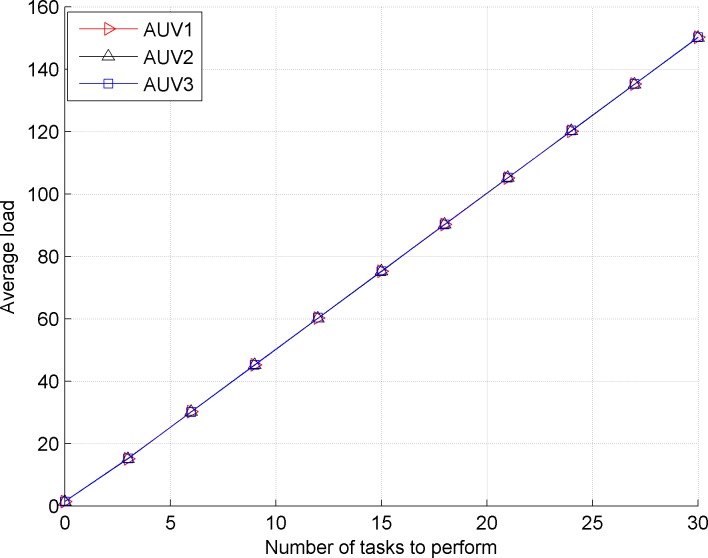
Average load of *AUV*_1_, *AUV*_2_ and *AUV*_3_ in the improved contract net model *AUV*_3_.

**Fig 4:**
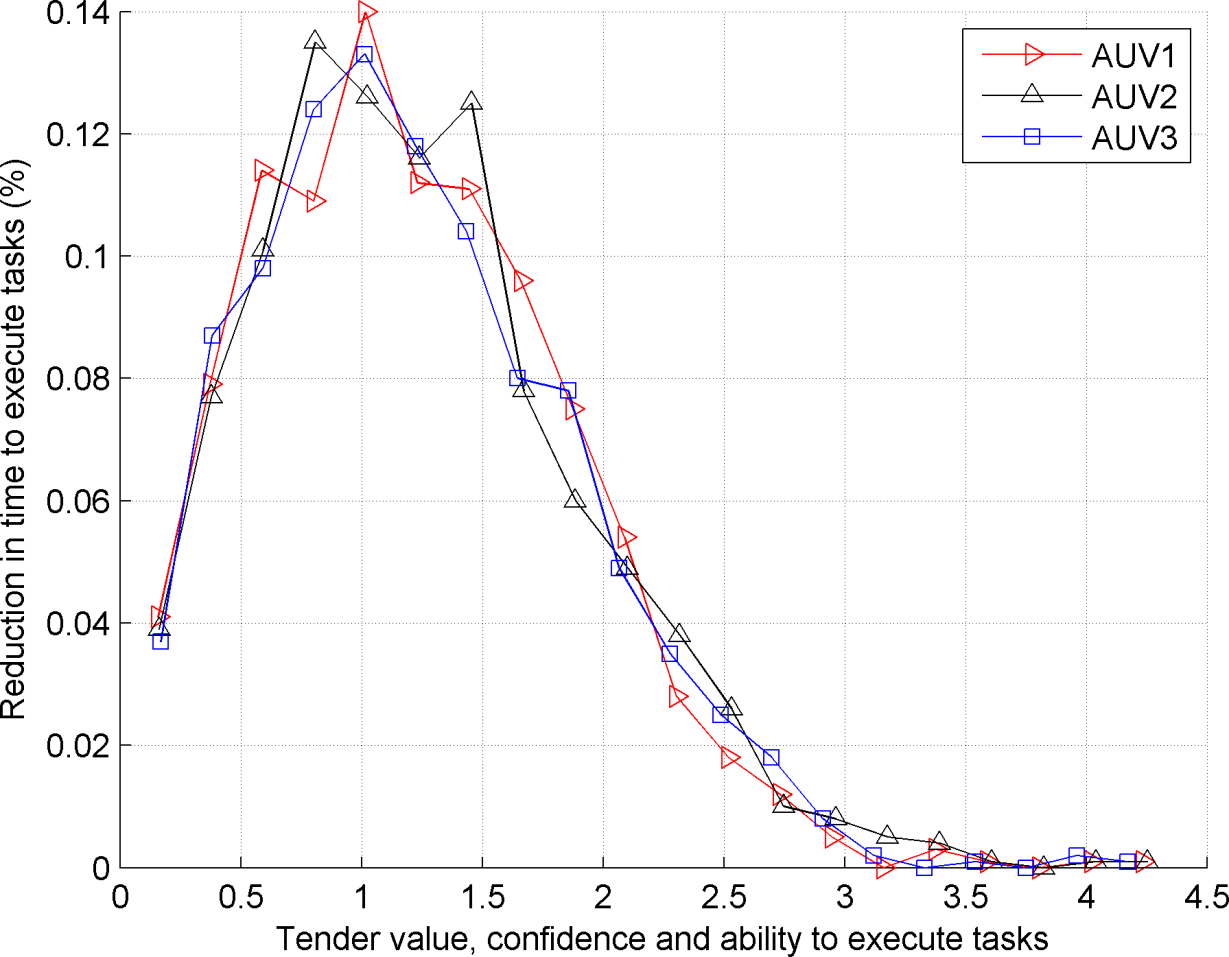
Reduced proportion (%) of *AUV*_1_, *AUV*_2_, and *AUV*_3_ tasks in the improved contract net model.

Fig 3 shows the average load of the AUVs in the contract net model based on the differential evolution quantum bee colony algorithm. Fig 4 shows the reduced proportion (%) of execution time in the contract net model with the introduced balance coefficient based on the differential evolution quantum bee colony algorithm.

Comparing the front and back images, we observe that the traditional contract net does not consider the load balance of the bidding AUV, which causes a large load difference for the bidding AUV. The improved contract net model satisfies the requirement of load balance because the proportions of load and task execution time of three bidding AUVs are basically equivalent.

**Fig 5:**
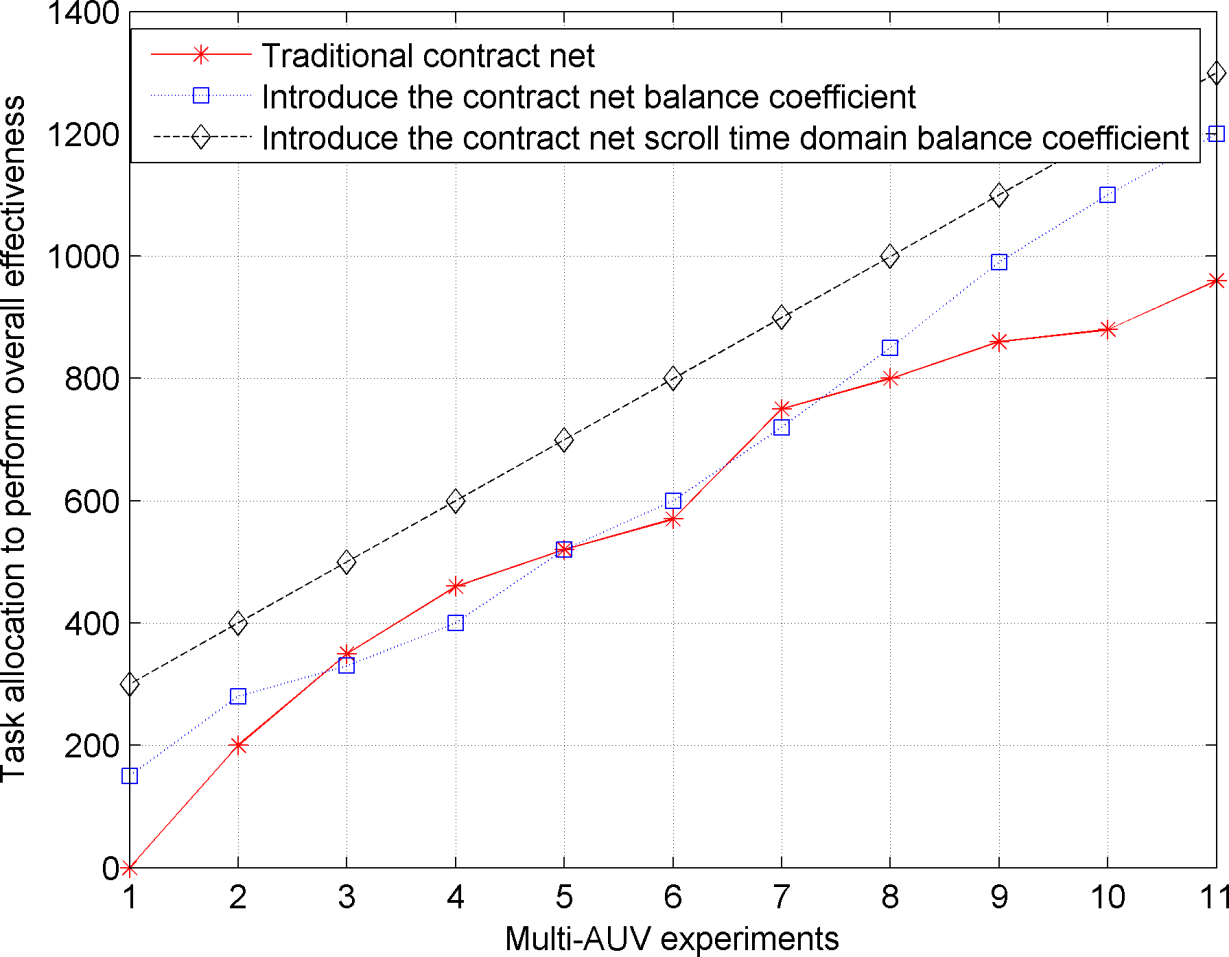
Comparison of the executive entirety effectiveness of the multi-AUV distributed task planning.

Fig 5 shows that the comparison of the executive entirety effectiveness of multiple AUVs in the distributed task planning experiment in the traditional contract net model and the contract net model with the introduced scroll time domain balance coefficient based on the quantum bee colony algorithm.

**Fig 6:**
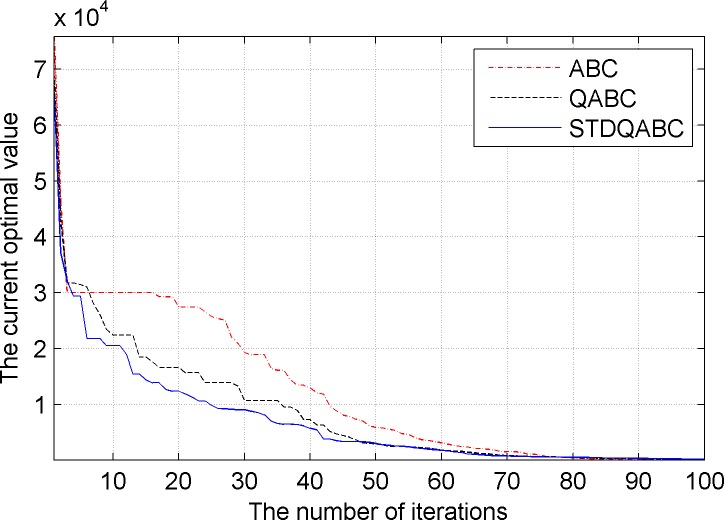
Comparison of the convergence performance of the ABC, QABC, and STDQABC algorithms.

Fig 6 shows the comparison of the convergence performance of the ABC, QABC, and STDQABC algorithms in the process of multi-AUV distributed task planning.

**Fig 7:**
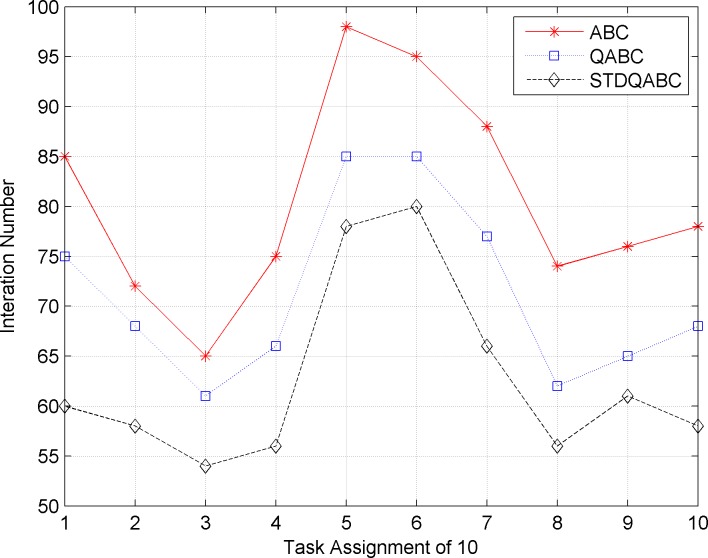
Comparison of the number of iterations for the ABC, QABC, and STDQABC algorithms.

**Fig 8:**
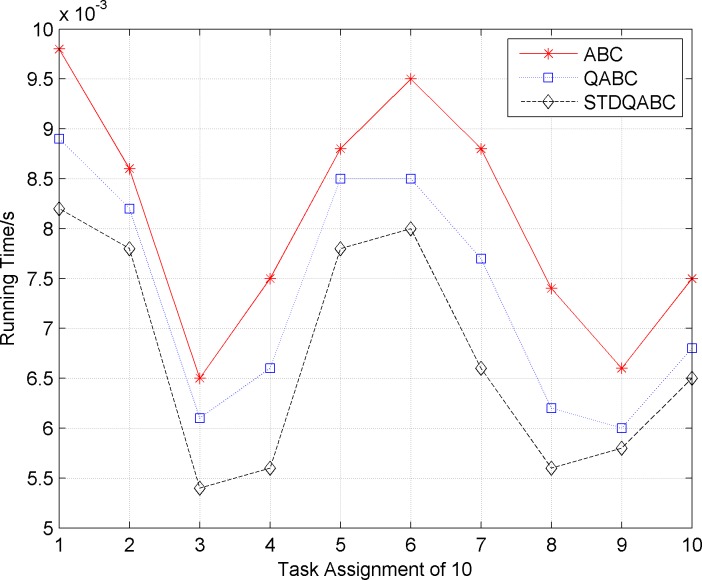
Comparison of the running time for the ABC, QABC, and STDQABC algorithms.

Figs 7 and 8 show the comparison of the number of iterations and running time when the ABC, QABC, and STDQABC algorithms are used to solve 10 task allocation cases to obtain the optimal solution.

**Fig 9:**
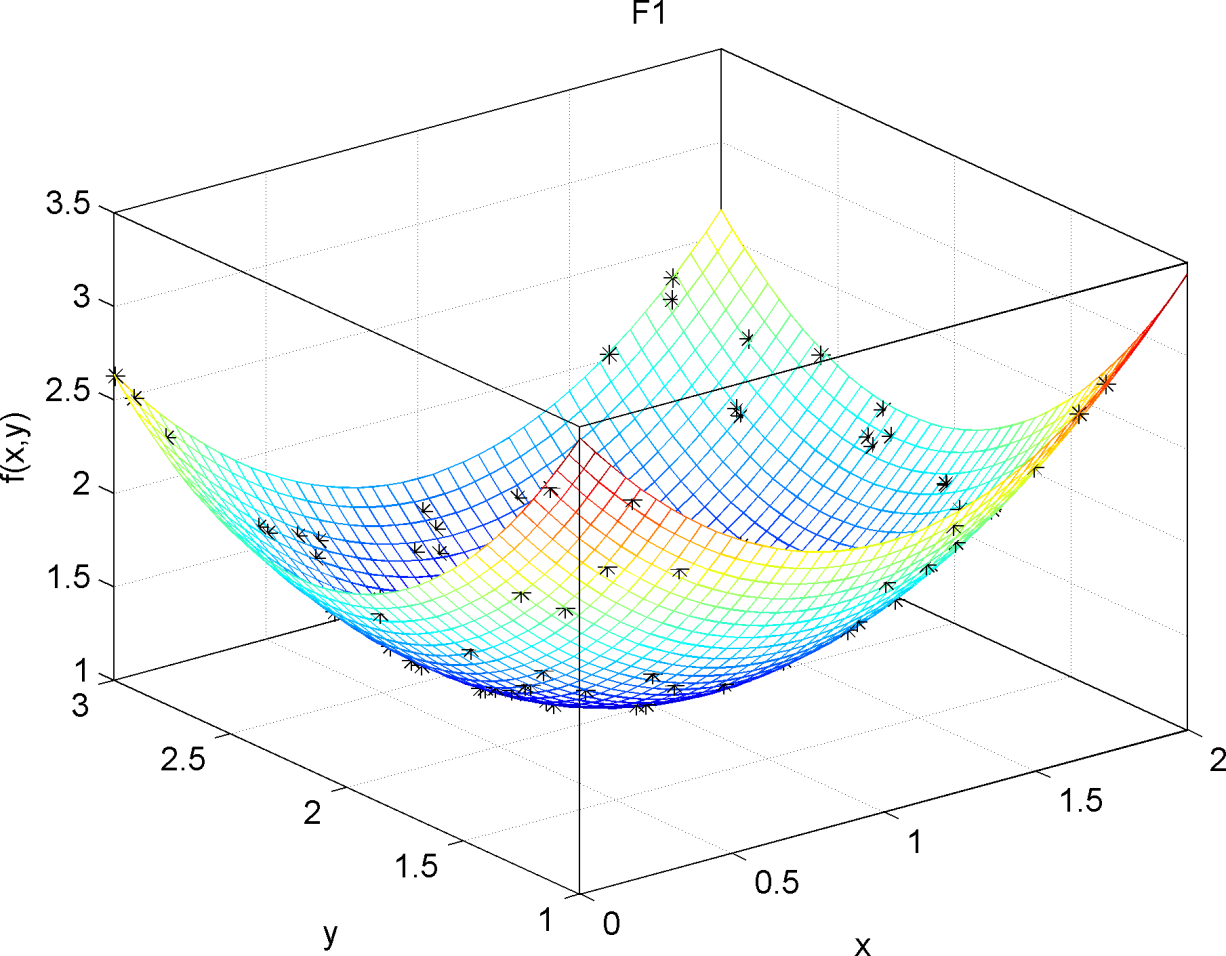
STDQABC algorithm single peak area test function F1 effect.

**Fig 10:**
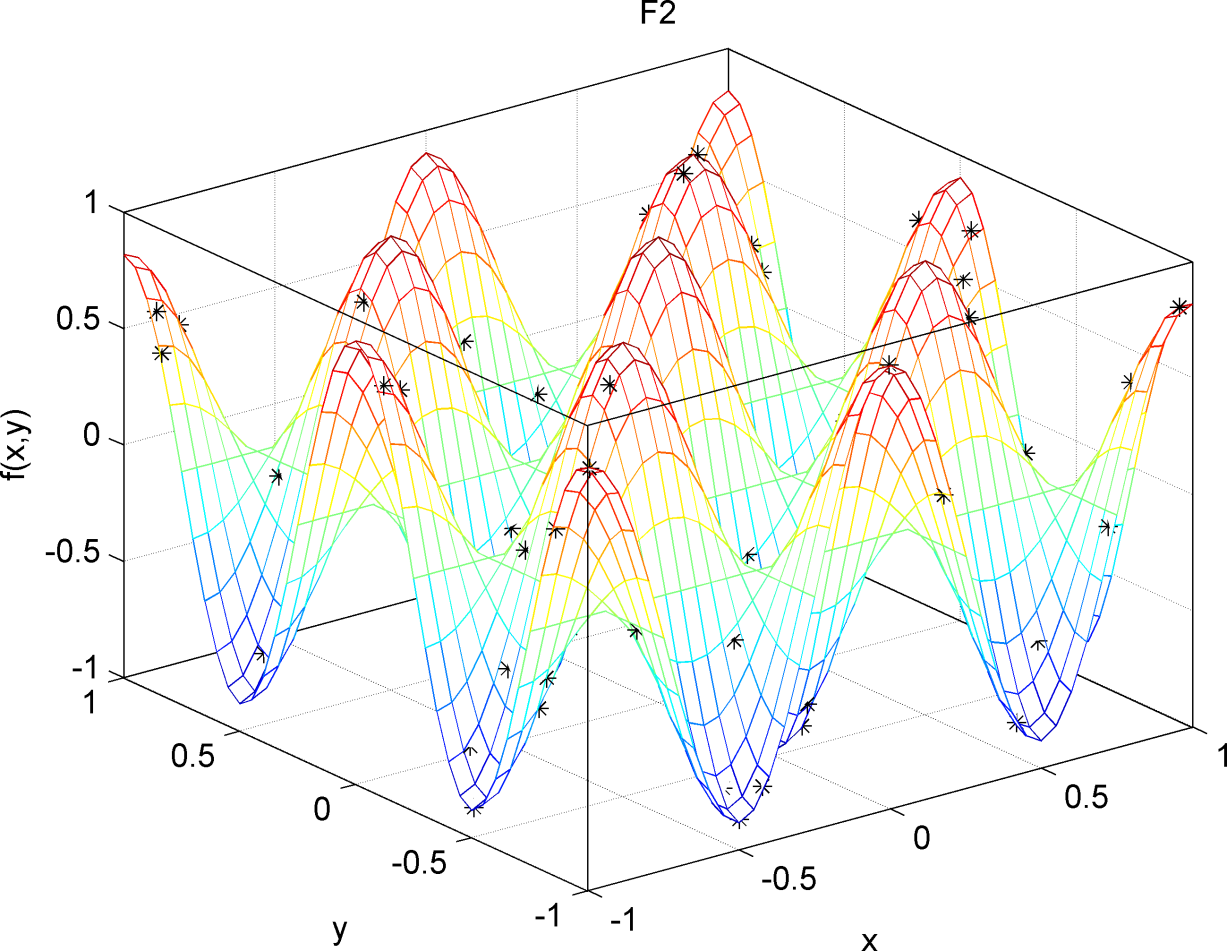
STDQABC algorithm multi-peak area test function F2 effect.

For the test functions with both unimodal and multi modal regions $F_{1}(x)=\sum_{i=1}^{n-1}\left[100{\left(x_{i+1}-x_{i}^{2}\right)}^{2}+{\left(x_{i}-1\right)}^{2}\right]$, DIM = 30, Range = [–100,100], fmin = 0 and $F_{2}(x)=-20\exp(-0.2\sqrt{\frac{1}{n}\sum_{i=1}^{n}x_{i}^{2}}-\exp(\frac{1}{n}\sum_{i=1}^{n}\cos(2\pi x_{i}))+20+e$, DIM = 30, Range = [–30,30], fmin = 0, Fig 9 and Fig 10 These results again demonstrate that DEQABC efficiently balances exploration and exploitation to approximate the global optimum.

The experimental results demonstrate that the STDQABC algorithm is faster than the QABC algorithm in terms of both number of iterations and running time and Get global optimal. Thus, the STDQABC algorithm can effectively improve the performance of the multi-AUV task planning. The STDQABC algorithm has faster and better stability than the ABC algorithm and STDQABC algorithm in solving the task planning problem for multiple AUVs and improving the system performance.

## Conclusion

In this paper, we propose a distributed task planning model based on the scroll time domain quantum bee colony algorithm to allow more rapid task planning for a greater number of AUVs and achieve global optimization in the multi-AUV distributed task planning. The balance coefficient is introduced to distribute the AUV task planning of the traditional contract net. The unbalanced load and other defects are improved in the multi-AUV distributed task planning of the traditional contract net. The scroll time domain quantum bee colony algorithm is applied to the process of multi-AUV dynamic distributed task planning in Uncertain Environment. The simulation experiment verifies that the quantum bee colony based on scroll time domain can avoid falling into local optima; shorten the convergence time; reduce the number of iterations; enhance the global, dynamic and adaptive capability of the bee colony algorithm; and effectively improve the overall performance of distributed task planning for multiple AUVs.

## Supporting information

S1 Appendix An overview of the process of the combinatorial model in mission scenario. (CSV)

S2 Appendix Productivity of the model in 10 mission scenarios. (CSV)
